# Factors Influencing Wild Venison Consumption in Illinois

**DOI:** 10.3390/ani15081132

**Published:** 2025-04-14

**Authors:** Huicheng Chen, Xiaohan Zhang, Junjie Wan, Craig A. Miller

**Affiliations:** 1 Joint International Research Laboratory of Catastrophe Simulation and Systemic Risk Governance, Beijing Normal University, Zhuhai 519087, China; 2 School of National Safety and Emergency Management, Beijing Normal University, Zhuhai 519087, China; 3 Chengdu NO. 7 YUCAI Middle School, Chengdu 610016, China; 4 Illinois Natural History Survey, University of Illinois at Urbana-Champaign, Champaign, IL 61820, USA; craigm@illinois.edu

**Keywords:** wild game consumption, influencing factors, Pearson correlation tests, logistic regression

## Abstract

Venison serves as a sustainable alternative to conventional protein sources and is closely tied to wildlife conservation efforts. This study sought to identify key factors influencing wild game consumption. Researchers conducted a self-administered mail-back survey targeting 5000 Illinois homeowners. Variables such as gender, dietary preferences, prior consumption experience, family involvement in hunting, and participation in outdoor activities were analyzed using Pearson correlation tests and logistic regression. The findings revealed that individuals who had previously consumed wild game, recently engaged in hunting or fishing, maintained a meat-rich diet, were male, or resided in rural areas were more likely to consume wild game. In conclusion, understanding these factors can help promote hunting as a sustainable food procurement method, raising awareness of its role in the food system and fostering greater societal acceptance of wild game meat.

## Simple Summary

The study investigated factors influencing wild venison consumption in Illinois. As a healthier and more environmentally sustainable alternative to conventional meat, understanding consumption patterns of wild game can contribute to wildlife conservation efforts. Through a survey of 5000 Illinois homeowners, the study revealed that individuals with prior experience eating venison, recent hunting or fishing activity, a preference for meat, male respondents, and those from rural areas were more likely to consume wild game. These findings can support efforts to promote hunting as a sustainable food procurement method, raising awareness of its role in the food system and fostering greater societal acceptance of wild game meat.

## 1. Introduction

The consumption of meat from legally harvested game animals carries significant cultural and social value. Scientific evidence supports the notion that wild game meat serves as a desirable alternative to conventional protein sources like pork, lamb, and beef [1,2,3,4]. Beyond its nutritional benefits, the consumption of wild game is intertwined with social relationships and plays a key role in fostering positive attitudes toward hunting [5,6,7,8]. Additionally, it is closely linked to support for wildlife conservation initiatives [9,10].

Despite its advantages, currently, the wild game meat market remains niche. Wild game meat is primarily sourced through hunting (e.g., NSSF reports hunters donated 11 million venison meals annually via food banks), but lacks large-scale commercial distribution compared to farmed meats. Consumption is higher in rural areas and among hunters, anglers, and white males. Urban and minority groups show lower engagement [11,12].

Under these circumstances, it is crucial to understand consumers’ behaviors regarding wild game consumption, and the factors that effectively promote the consumption of wild game.

## 2. Materials and Methods

Previous studies have indicated that consumers’ preferences for wild game consumption may be influenced by personal experiences, family influences, cultural factors, and environmental context [13,14,15,16]. Given the close connection between outdoor activities like hunting and fishing and wild game consumption, the willingness to engage in such activities may also reflect consumers’ acceptance of wild game. Based on this, we hypothesized that the inclination to consume wild game could be shaped by factors such as prior experience, dietary habits, family background, community context, gender, and participation in outdoor recreation.

Data were collected through a random sample of 5000 Illinois homeowners. Homeowner names and addresses were provided via public property tax records. Data were collected using three waves of a self-administered mail-back questionnaire. On 4 September 2018, participants received an 8-page questionnaire, a cover letter, and a postage-paid return envelope. A thank you/reminder postcard was mailed to recipients on 19 September 2018. Non-respondents were mailed a second questionnaire and cover letter on 1 October 2018, followed by a second postcard mailing on 17 October 2018. A third and final questionnaire and cover letter were mailed to non-respondents on 30 October 2018. We deleted 178 subjects due to incorrect addresses and received a total of 1367 completed questionnaires for a 28% response rate.

To analyze the data, we employed Pearson correlation tests, binary logistic regression, and multinomial logistic regression to examine the relationships between the inclination to consume wild game and hypothesized influencing factors such as gender, dietary preferences, past experience with wild game consumption, upbringing and residential background, family involvement in hunting, and recent participation in outdoor activities. The inclination to consume wild game (the dependent variable of the logistic regression) was derived from “If you had the opportunity, would you eat wild meat?”. Among them, “1” represents having eaten wild game, and “0” represents not having eaten wild game. The hypothesized influencing factors (the independent variable) were derived from the 8 questions shown in Table 1.

## 3. Results

The inclination to consume wild game was measured using responses to the question, “If you had the opportunity, would you eat wild meat?” Independent variables were derived from corresponding questions (detailed in Table 1). Results (presented in Table 2) showed no significant collinearity among the variables, and multinomial logistic regression analysis further clarified their impact patterns (Table 3).

The “Sig.” values of the Pearson Chi-Square tests (presented in Table 2) for multiple variables in the table are all less than 0.05. Variables (presented in Table 2) are significantly correlated with “If you had the opportunity, would you eat wild meat?”. These results imply that these factors exhibit obvious differences and regularities in the decision-making regarding whether to consume wild game meat.

In both the binary and multinomial logistic regression analyses, the B values of both “Have you ever eaten wild game meat obtained through hunting?” and “Do you or any members of your immediate family currently hunt?” are positive (Sig. < 0.05), indicating that these independent variables have a significant positive impact on the dependent variable.

In the binary logistic regression analysis, dietary type (“B” = 0.835, “Sig.” < 0.05), suggests a significant positive impact on the willingness to consume wild game meat. However, the multinomial logistic regression regression (Sig. = 0.454 > 0.05), indicates no statistically significant influence on the dependent variable.

In the binary regression analysis, both outdoor activities show positive B—values (Sig. < 0.05), indicating a significant positive impact on the dependent variable. In the multinomial logistic regression analysis, fishing (Sig. < 0.05) remains significant, but hunting’s (Sig. > 0.05) impact on the dependent variable is insignificant in explaining changes.

In both regression analyses, the “B” values of gender are negative (Sig. < 0.05), indicating that women are relatively less willing to consume wild game meat compared to men. The gender difference is quite prominent in terms of the willingness to consume wild meat.

In the binary regression analysis, the “B” values of both current and growing-up community sizes are negative (Sig. < 0.05), suggesting urban communities correlate with a lower likelihood of consuming wild game meat and significant impact. However, the multinomial logistic regression indicates that community type (Sig. > 0.05) has no significant impact on the dependent variable.

In the collinearity diagnosis, the tolerance of all independent variables ranges from 0.694 to 0.919, and the variance inflation factor ranges from 1.088 to 1.442. Therefore, there is no severe collinearity among the independent variables in this model, and the validity and stability of the model are not affected by the high correlation between the independent variables.

## 4. Discussions

Our study found that recent participation in hunting or fishing activities was positively associated with a greater willingness to consume wild game. This correlation aligns with findings from a similar study conducted in Italy [17], suggesting that an affinity for hunting and related outdoor activities often translates into a preference for game meat. We also speculate that individuals who frequently engage in outdoor activities may be more open to trying new experiences, including the consumption of wild game.

A less commonly discussed factor in previous studies is the role of dietary preferences. Our findings indicate that individuals with a meat-rich diet are more likely to try wild game. In Illinois, women were less willing to consume wild game compared to men, a trend consistent with findings from other cultures, such as Sweden [3,18]. Literature suggests that wild game consumption is more strongly associated with masculine traits, particularly among specific consumer groups like hunters [19].

Additionally, individuals born and residing in rural areas demonstrated a stronger inclination toward wild game consumption compared to their urban counterparts, a pattern also observed in Sweden [2]. Rural environments often provide greater access to hunting and agricultural activities, which may foster a stronger connection to wild game. Research by Heberlein, Ericsson, and Wollscheid [20] supports this, identifying rural residence as one of the strongest predictors of hunting participation. Moreover, individuals with family members recently involved in hunting were more likely to consume wild game, highlighting the influence of upbringing and environmental exposure on consumption patterns [13]. These findings underscore the role of cultural and environmental factors in shaping attitudes toward wild game consumption.

This study provides insights for advancing sustainable food systems, wildlife conservation, and societal engagement with wild game consumption. Beyond validating hypothesized relationships between influencing factors and the inclination to consume wild game, our results highlight three practical pathways for application.

First, the link between rural residency, family influence, and wild game consumption suggests the potential of community-based interventions. Localized campaigns help to normalize game meat in mainstream diets, such as workshops on hunting skills, cooking demonstrations, etc. Partnerships with schools, farmers’ markets, and conservation organizations could integrate wild game into local food networks, bridging urban–rural divides and fostering cross-cultural acceptance. However, any efforts to commercialize wild game must be carefully balanced against ecological risks. Historical precedents demonstrate that unregulated wildlife markets can lead to overexploitation and regional extinctions—particularly for slow-reproducing or habitat-sensitive species. Thus, scaling up wild game consumption demands parallel investments in science-based monitoring (e.g., population viability analyses), adaptive harvest quotas, and traceability mechanisms to ensure that market incentives align with conservation goals.

Second, the persistent gender gap in wild game acceptance calls for gender-sensitive strategies. Marketing campaigns could highlight the health benefits of lean game meat (e.g., lower saturated fat) or its role in eco-conscious lifestyles, appealing to female audiences.

Third, recreational hunting’s dual role as a food source and conservation tool offers unique policy opportunities. Governments and NGOs could create incentives, such as tax rebates for hunters donating game meat to food banks or certifications for restaurants using sustainably harvested wild game. These measures would address food insecurity while boosting public recognition of hunting’s socioeconomic value. Integrating wild game into national dietary guidelines as a climate-friendly protein could also align individual choices with environmental goals.

This study quantitatively analyzed the factors influencing wild game consumption. However, further study is necessary to explore the real motivations behind such consumption. More surveys to investigate the true motivation, comparative analyses across regions with diverse cultural norms, and longitudinal studies tracking how exposure to hunting culture shifts urban consumers’ attitudes over time would further deepen our understanding of wild game consumption.

In summary, this research offers a roadmap to transform wild game from a niche dietary choice into a cornerstone of sustainable food systems. By aligning community traditions, targeted marketing, and policy innovation, stakeholders can amplify the ecological and cultural benefits of wild game consumption while addressing conservation and food sustainability challenges.

## 5. Conclusions

This study investigated factors influencing wild game consumption to assess its potential as a sustainable protein source linked to wildlife conservation. Through a survey of 5000 Illinois homeowners, the study analyzed variables including demographics, dietary habits, and outdoor activities using Pearson correlations and logistic regression. Key findings indicated that individuals more likely to consume wild game were male, resided in rural areas, had prior experience eating wild game, participated in recent hunting or fishing activities, or maintained meat-centric diets. The results underscore the importance of targeted outreach to promote wild game as an environmentally conscious food choice. Policymakers and conservation advocates can enhance public acceptance of hunting’s role in sustainable food systems and wildlife management, fostering broader societal engagement with eco-friendly dietary alternatives.

## Figures and Tables

**Table 1:** The proportion of responses to each option for related questions regarding the independent and dependent variables of this study. (*n* = 1367).

Description		Questions	Option	Percentage
Inclination to consume wild game (Dependent Variable)		If you had the opportunity, would you eat wild meat?	Yes	63.60%
			No	36.40%
Factors may influence the inclination (Independent Variables)	dietary habits	Which of the following categories best describes your diet?	Other diet	73.70%
			My meals revolve around meat every day	26.30%
	prior experience	Have you ever eaten wild game meat obtained through hunting?	No	26.90%
			Yes	71.90%
			I am not sure	1.20%
	family background	Do you or any members of your immediate family currently hunt?	No	56.20%
			Yes	43.80%
	gender	What is your gender?	Male	55.00%
			Female	45.00%
	community context	How would you describe the size of your community where you live now?	Urban	81.30%
			Rural	18.70%
		How would you describe the size of your community where you grew up?	Urban	80.40%
			Rural	19.60%
	participation in outdoor recreation	Which of the following outdoor recreation activities have you participated in during the past 12 months: Fishing	No	60.40%
			Yes	39.60%
		Which of the following outdoor recreation activities have you participated in during the past 12 months: Hunting	No	85.30%
			Yes	14.70%

**Table 2:** Pearson correlation tests and binary logistic regression results. The dependent variable was derived from the question “If you had the opportunity, would you eat wild meat?” (*n* = 1367).

Independent Variables Were Derived from These Questions.	Chi-Square Tests (Pearson Chi-Square)		Logistic Regression		
	Value	Sig. *	B	Wald	Sig.
Have you ever eaten wild game meat obtained through hunting?	122.255	**0.** **000**	0.275	100.477	**0.** **000**
Which of the following categories best describes your diet?	3.994	**0.** **000**	0.835	13.571	**0.** **000**
Do you or any members of your immediate family currently hunt?	78.034	**0.** **000**	1.824	70.077	**0.** **000**
How would you describe the size of your community where you live now?	5.195	**0.** **023**	−0.565	5.110	**0.** **024**
How would you describe the size of your community where you grew up?	12.992	**0.** **004**	−0.918	12.429	**0.** **000**
What is your gender?	43.440	**0.** **000**	−1.227	41.836	**0.** **000**
Which of the following outdoor recreation activities have you participated in during the past 12 months: Fishing	62.643	**0.** **000**	1.662	56.792	**0.** **000**
Which of the following outdoor recreation activities have you participated in during the past 12 months: Hunting	44.496	**0.** **000**	3.309	20.960	**0.** **000**

* The significant results (Sig. ≤ 0.05) are presented in bold.

**Table 3:** Multinomial logistic regression analysis results. The dependent variable was derived from the question “If you had the opportunity, would you eat wild meat?” (*n* = 1367).

Independent Variables Were Derived from These Questions.	Unstandardized Coefficients		Standardized Coefficients	t	Sig. *	Collinearity Statistics	
	B	Std. Error	Beta			Tolerance	VIF
(Constant)	0.615	0.143		4.303	**0.000**		
Which of the following categories best describes your diet?	−0.031	0.041	−0.028	−0.749	0.454	0.919	1.088
Have you ever eaten wild game meat obtained through hunting?	0.348	0.041	0.336	8.412	**0.000**	0.814	1.228
Do you or any members of your immediate family currently hunt?	0.165	0.042	0.171	3.971	**0.000**	0.706	1.417
What is your gender?	−0.164	0.038	−0.170	−4.319	**0.000**	0.839	1.192
How would you describe the size of your community where you live now?	0.029	0.051	0.023	0.566	0.572	0.766	1.306
How would you describe the size of your community where you grew up?	−0.068	0.050	−0.056	−1.364	0.173	0.764	1.309
Which of the following outdoor recreation activities have you participated in during the past 12 months: Fishing	0.130	0.040	0.133	3.254	**0.001**	0.779	1.283
Which of the following outdoor recreation activities have you participated in during the past 12 months: Hunting	0.033	0.059	0.024	0.557	0.578	0.694	1.442

* The significant results (Sig. ≤ 0.05) are presented bold.

## Data Availability

Restrictions apply to the availability of these data. Data were obtained from the Illinois Natural History Survey and are available from the corresponding authors with the permission of the Illinois Natural History Survey.
